# Glucocorticoid Receptor Knockdown Decreases the Antioxidant Protection of B16 Melanoma Cells: An Endocrine System-Related Mechanism that Compromises Metastatic Cell Resistance to Vascular Endothelium-Induced Tumor Cytotoxicity

**DOI:** 10.1371/journal.pone.0096466

**Published:** 2014-05-06

**Authors:** Elena Obrador, Soraya L. Valles, María Benlloch, J. Antoni Sirerol, José A. Pellicer, Javier Alcácer, Javier Alcácer-F. Coronado, José M. Estrela

**Affiliations:** 1 Department of Physiology, University of Valencia, Valencia, Spain; 2 Faculty of Experimental Sciences, San Vicente Martir Catholic University, Valencia, Spain; 3 Pathology Laboratory, Quirón Hospital, Valencia, Spain; Istituto Superiore di Sanità, Italy

## Abstract

We previously reported an interorgan system in which stress-related hormones (corticosterone and noradrenaline), interleukin-6, and glutathione (GSH) coordinately regulate metastatic growth of highly aggressive B16-F10 melanoma cells. Corticosterone, at levels measured in tumor-bearing mice, also induces apoptotic cell death in metastatic cells with low GSH content. In the present study we explored the potential role of glucocorticoids in the regulation of metastatic cell death/survival during the early stages of organ invasion. Glucocorticoid receptor (GCR) knockdown decreased the expression and activity of γ-glutamylcysteine synthetase (γ-GCS), the rate-limiting step in GSH synthesis, in metastatic cells *in vivo* independent of the tumor location (liver, lung, or subcutaneous). The decrease in γ-GCS activity was associated with lower intracellular GSH levels. Nrf2- and p53-dependent down-regulation of γ-GCS was associated with a decrease in the activities of superoxide dismutase 1 and 2, catalase, glutathione peroxidase, and glutathione reductase, but not of the O_2_^−^-generating NADPH oxidase. The GCR knockdown-induced decrease in antioxidant protection caused a drastic decrease in the survival of metastatic cells during their interaction with endothelial cells, both *in vitro* and *in vivo*; only 10% of cancer cells attached to the endothelium survived compared to 90% survival observed in the controls. This very low rate of metastatic cell survival was partially increased (up to 52%) *in vivo* by inoculating B16-F10 cells preloaded with GSH ester, which enters the cell and delivers free GSH. Taken together, our results indicate that glucocorticoid signaling influences the survival of metastatic cells during their interaction with the vascular endothelium.

## Introduction

Glutathione (γ-glutamyl-cysteinyl-glycine, GSH), due to its reactivity and high intracellular concentrations (up to 10 mM in the liver and in various highly malignant cells), is involved in many cellular functions. GSH is particularly relevant in cancer cells as it is involved in regulating e.g. carcinogenic mechanisms, growth and dissemination, and multidrug and radiation resistance [1], [2], [3]. A classical model in metastasis research, the highly metastatic B16 melanoma F10 (B16-F10), shows higher GSH content, GSH synthesis rate, and lower GSH efflux than the B16-F1 cell subset with low metastatic potential [4].

Interleukin 6 (IL-6) (mainly of tumor origin) facilitates GSH release from hepatocytes and its interorgan transport through the blood circulation to growing metastatic foci in B16-F10-bearing mice [5]. Recently we studied if the capacity of metastatic cells to overproduce IL-6 is regulated by cancer cell-independent mechanisms. We found that pathophysiological levels of stress-related hormones (corticosterone and noradrenaline) increase the expression and secretion of IL-6 in B16-F10 cells [6]. *In vitro* experiments showed that corticosterone, but not noradrenaline, also induces mitochondria-dependent apoptotic cell death in B16-F10 cells with low GSH content [6]. Indeed the intracellular thiol redox state, controlled by GSH, is one of the endogenous effectors involved in regulating the activation of cell death pathways [7]. Mitochondrial GSH (mtGSH) oxidation, in particular, facilitates opening of the mitochondrial permeability transition pore complex, a causal factor in the mitochondrion-based mechanism that leads to cell death [3]. The corticosterone-induced increase in reactive oxygen species (ROS) generation contributes to mtGSH depletion and activation of apoptosis [6]. However, B16-F10 cells with high GSH content were found resistant to corticosterone-induced cell death [6].

Glucocorticoids have been widely used in cancer, in conjunction with other treatments, because (in addition to other potential benefits) they have proapoptotic properties in different cancer cell types. These hormones may also induce a yet undefined resistant phenotype, thereby facilitating fast growth and metastasis of different solid tumors [8], [9]. Under *in vivo* conditions, due to natural tumor heterogeneity [10], we must expect different metastatic cell subsets with different GSH content [2]. Because glucocorticoids are able to increase ROS generation [6], surviving metastatic cells may activate adaptations in GSH metabolism as well as in other oxidative stress-related molecular systems. The ability of cancer cells to dynamically adapt, evading our physiological defense systems and resisting anticancer therapies, is emerging as a key feature of malignant behavior [11], [12], [13], [14], [15].

In the present study we explored possible links among glucocorticoids, GSH, oxidative stress, and the survival of metastatic cells using glucocorticoid receptor knockdown. We found lower antioxidant protection in metastatic cells in the absence of glucocorticoid signaling, thus leading to an increase in vascular endothelium-induced tumor cytotoxicity.

## Materials and Methods

### B16-F10 and iB16 melanoma cell culture

Murine B16-F10 (ATCC, Rockville, MD) or iB16 cells were cultured in Dulbecco's modified Eagle's medium (DMEM, Life Technologies, Alcobendas, Spain), pH 7.4, supplemented with 10% fetal calf serum (Life Technologies), 10 mM HEPES, 40 mM NaHCO_3_, 100 units/ml penicillin, and 100 µg/ml streptomycin [16]. Cell integrity was assessed by trypan blue exclusion and the leakage of lactate dehydrogenase activity [16].

### Animals

Syngenic male C57BL/6J mice (12 weeks old) from Charles River Laboratories (Barcelona, Spain) were fed a standard diet (Letica, Barcelona, Spain) *ad libitum*. Mice were kept on a 12-h light/12-h dark cycle with the room temperature maintained at 22°C. Procedures involving animals were in compliance with international laws and policies (EEC Directive 86/609 and National Institutes of Health guidelines).The protocol was approved by the Committee on the Ethics of Animal Experiments of the University of Valencia (Spain). All surgery was performed under sodium pentobarbital anesthesia, and all efforts were made to minimize suffering.

### Local tumor growth

B16-F10 cells were harvested from culture flasks using 2 mM EDTA for 5 min at 37°C, washed twice in DMEM, resuspended in the same culture medium, and injected into the foot pad of the right hind-limb (10^4^ cells/20 µl) of the C57BL/6J mice. Local tumor growth was determined by measuring foot pad diameter with calipers every 2 days. Tumor size was calculated according to the following formula: tumor diameter  =  diameter of foot pad with growing tumor - diameter of DMEM-treated contralateral foot pad.

### Experimental metastases

Hepatic and lung metastases were produced by intravenous injection of 10^5^ viable B16-F10 cells (suspended in 0.2 ml of DMEM) into the portal and tail veins, respectively, of anesthetized mice (Nembutal, 50 mg/kg intraperitoneally). Mice were cervically dislocated 10 days after tumor cell inoculation. Livers and lungs were fixed with 4% formaldehyde in PBS (pH 7.4) for 24 h at 4°C and then embedded in paraffin. Metastasis volume (i.e., mean percentage of organ volume occupied by metastases) was determined as described previously [17].

### Isolation of iB16 cells *in vivo*

Anti-Met-72 monoclonal antibodies and flow cytometry-coupled cell sorting were used, as previously described [11], [18], to isolate viable melanoma cells from the tumor growing in the foot pad or from metastatic foci. Anti-Met-72 monoclonal antibodies, which react with a 72-kDa cell-surface protein (Met-72) expressed at high density on B16 clones with high metastatic activity, were produced as previously described [19]. Melanoma cells were separated by fluorescence-activated cell sorting, using a MoFlo High-Performance Cell Sorter (DAKO, Copenhagen, Denmark), and collected into individual chambered tissue culture slides (NalgeNunc International Corp., Penfield, NY). The sorted tumor cells were harvested and plated in 25-cm^2^ polystyrene flasks (Falcon Labware) as described above.

### Determination of GSH and GSSG

GSH and glutathione disulfide (GSSG) levels were determined by liquid chromatography-mass spectrometry using a Quattro micro^TM^triplequadrupole mass spectrometer (Micromass, Manchester, UK) equipped with a Shimadzu LC-10ADVP pump and a SCL-10AVP controller system with an SIL-10ADVP autoinjector (Shimadzu Corp., Kyoto, Japan) following procedures previously described [20]. Tissue sample collection and processing were performed according to published methodology [21] in which rapid N-ethylmaleimide derivatization was used to prevent GSH auto-oxidation.

### GSH synthesis

To measure GSH synthesis rates, cultured cells were harvested 24 h after seeding, washed twice, re-suspended in ice-cold Krebs-Henseleit bicarbonate medium (pH 7.4), and incubated (5 mg dry weight/ml) in 10-ml Erlenmeyer flasks (final volume 2 ml) for 60 min at 37°C in the presence of amino acid precursors (5 mM L-Gln, 2 mM Gly, 1 mM L-Ser, 1 mM N-acetylcysteine). Glucose (5 mM) and bovine serum albumin (2%) were always present. GSH synthesis was calculated from the total GSH content after 0, 20, 40, and 60 min of incubation. GSH efflux was calculated from the total glutathione (GSH + 2xGSSG) and GSSG content in the culture medium at 0, 30, 60, and 120 min (starting 24 h after seeding).

### Enzyme assays

To measure enzyme activity, isolated tumor cells were homogenized in 0.1 M phosphate buffer (pH 7.2) at 4°C [17]. γ-Glutamylcysteine synthetase (γ-GCS) and GSH synthetase (GSH-S) activities were measured as described previously [16]. Superoxide dismutase (SOD) activity was measured as described by Flohé and Otting [22] using 2 mM cyanide in the assay medium to distinguish mangano-type enzyme (SOD2) from the cuprozinc type (SOD1). Catalase (CAT) activity was analyzed as described by Aebi [23]. Glutathione peroxidase (selenium-dependent, GPX) activity was measured as described by Flohé and Gunzler [24] using H_2_O_2_ as a substrate. Glutathione reductase (GR) activity was determined as described by Akerboom and Sies [25]. γ-Glutamyl transpeptidase (γ-GT) activity was measured as described previously [26]. NADPH oxidase (NOX) activity was measured by chemiluminescence following the methodology of Wind et al. [27]. Protein concentration was determined with the Pierce BCA protein assay (Fisher Scientific, Waltham. MA).

### Measurement of adrenocorticotropin hormone and corticosterone levels

Plasma levels of ACTH (Calbiotech, Inc., Spring Valley, CA) and corticosterone (Kamiyama Biomedical Co., Seattle, WA) were quantified by ELISA according to the instructions of the suppliers.

### Measurement of IL-6 levels

Blood samples were centrifuged at 14000 rpm for 10 min at 4°C to separate the serum. Concentration of IL-6 in the serum was determined using commercially available mouse cytokine ELISA kits from Innovative Research (Novi, MI).

### Glucocorticoid receptor knockdown: lentivirus production, titering, and transduction of target cell lines

HEK-293T cells (ATCC) used for lentiviral production were grown in DMEM containing 10% FBS, 4.5 g/l glucose, 50 U/ml penicillin, 50 mg/ml streptomycin, 1 mM sodium pyruvate, 4 mM L-glutamine, and 0.1 mM non-essential amino acids. The LENTI-Smart system from InvivoGen (San Diego, CA) was used according to the manufacturer's protocol. Following transduction with integrating lentiviral vectors, the transgene is integrated into the target cell genome to obtain stable transgene expression. Briefly, HEK-293T cells were plated in T-75 cm^2^ flasks at a density of 10×10^6^ cells/plate. Twenty-four hours following the initial plating, the culture medium was aspirated and replaced with serum-free medium containing the transfection mixture of LENTI-Smart and the transfer plasmid containing the specific gene sequence. This sequence is directed against a consensus sequence of the mouse (*Mus musculus*) glucocorticoid receptor (GCR) and designed from the Ensemble genome browser/database (www.ensembl.org): flanking 5′ region, GATCCCC; shRNA sequence (passenger), CAGACTTTCGGCTTCTGGA; hairpin, TTCAAGAGA; reverse shRNA sequence (guide), TCCAGAAGCCGAAAGTCTG; flanking 3′ region, TTTTTGGAAA. Cells transfected with lentiviral vector not harboring any gene (InvivoGen) were used as a negative control. The transfection duration in serum-free medium was 6 h, followed by replacement of the serum-free medium + transfection reagents with fresh serum-containing medium. Lentiviral particles were collected 48 h after transfection. Cell/viral debris was removed from the collected supernatants by centrifugation (2000 rpm ×5 min) and filtration using a 0.45 mm PVDF (low protein binding) filter. Lentiviral particles were concentrated by ultracentrifugation (70000 x g for 2 h at 4°C). The fibrosarcoma cell line HT1080 (ATCC) was used as the “gold standard” for titering lentiviral vectors using the green fluorescent protein (GFP) within the transfer construct as a marker for microscopic analysis. Lentiviral vectors contain the viral capsid protein (p24), which is encoded by the gag gene. Thus, an ELISA was used to determine the amount of p24 in the supernatant as it is directly proportional to the amount of lentiviral vector. Forty-eight hours following viral transduction, the number of GFP-positive colonies per well was counted by fluorescence microscopy. Transducing units per milliliter was calculated as follows: (T x V)/N, where T is the titer of the lentiviral vector stock, V is the volume of lentiviral vectors (in ml), and N is the number of cells to be transduced. B16 melanoma cell variants were transduced and subsequently selected by puromycin treatment to produce B16-shGCR cell variants. Clonal populations of each cell line were obtained by flow cytometric cell sorting based on GFP positivity using the MoFlo High-Performance Cell Sorter. Cells transfected with retroviral vector harboring the GFP gene were used as a negative control. Established clones were grown as described above in medium supplemented with 0.5 mg/ml puromycin. Silencing was confirmed by immunoblotting. The anti-mouse GCR monoclonal antibodies were purchased from Santa Cruz Biotechnology (Santa Cruz, CA).

### RT-PCR and detection of mRNA

Total RNA was isolated using the TRIzol kit from Invitrogen (Gaithersburg, MD) following the manufacturer's instructions. cDNA was obtained using a random hexamer primer and a MultiScribe Reverse Transcriptase kit as recommended by the manufacturer (TaqMan RT Reagents, Applied Biosystems, Foster City, CA). PCR master mix and AmpliTaq Gold DNA polymerase (Applied Biosystems) were added to the specific primers (Sigma-Genosys) previously reported for the γ-GCS subunits (heavy, γ-GCS-HS; light, γ-GCS-LS) [28] and glyceraldehyde-3P-dehydrogenase (GAPDH) [16]. Primers for the antioxidant enzyme activities (Sigma-Genosys) were: SOD1 (forward, 5′-TGGGTTCCACGTCCATCAG-3′; reverse, 5′-ACACCGTCCTTTCCAGCAG-3′), SOD2 (forward, 5′-ATGCAGCTGCACCACAGCAA-3′; reverse, 5′-ACTTCAGTGCAGGCTGAAGAG-3′), CAT (forward, 5′-ATGGTCTGGGACTTCTGGAGTCTTC-3′; reverse, 5′-GTTTCCTCTCCTCCTCATTCAACAC-3′), GPX (forward, 5′-GGGACTACACCGAGATGAACGA-3′; reverse, 5′-ACCATTCACTTCGCACTTCTCA-3′), GR (forward, 5′-GGAAGTCAACGGGAAGAAGTTCACTG-3′; reverse, 5′-CAATGTAACCGGCACCCACAATAAC-3′), and NOX (p22phox) (forward, 5′-GGCACCATCAAGCAACCACC-3′; reverse, 5′-CTCATCTGTCACTGGCATTGGG-3′). Real-time quantification of mRNA relative to GAPDH was performed with a SYBR Green I assay and an iCycler detection system (Biorad, Hercules, CA). Target cDNA was amplified using the following conditions: 10 min at 95°C followed by 40 cycles of denaturation at 95°C for 30 sec and annealing and extension at 60°C for 1 min. Changes in fluorescence were measured in real time during the extension step. The threshold cycle (C_T_) was determined and the relative gene expression expressed as fold change  = 2^–Δ(Δ C^_T_^)^, where Δ C_T_  =  C_T_target – C_T_ GAPDH, and Δ (Δ C_T_)  =  Δ C_T_treated – Δ C_T_control.

### Nrf1 and Nrf2 measurements

The NE-PER extraction kit from Thermo Scientific (Rockford, IL) was used for nuclear protein extraction according to the manufacturer's instructions. The protein content was determined by the Bradford assay [29]. The antibodies (mouse monoclonal primary antibodies) against Nrf1 (nuclear respiratory factor 1) or Nrf2 (nuclear factor (erythroid-derived 2)-like 2) were purchased from Abcam (Cambridge, UK). A total of 50µg of protein was boiled in Laemmli buffer and resolved by 12.0% SDS-PAGE. Proteins were transferred to a nitrocellulose membrane (Hybond C-extra, GE Healthcare Europe GmbH, Barcelona, Spain) and subjected to Western blotting. The blotted membrane was blocked for 1 h at room temperature in Tris-buffered saline (TBS) containing 5% (w/v) membrane-blocking reagent (non-fat dried milk). All antibody incubations were carried out at room temperature in TBS containing 1% membrane-blocking reagent. The incubation steps were followed by three washing steps of 5 min using TBS containing 0.1% Tween 20. The blots were developed using horseradish peroxidase-conjugated secondary antibody and enhanced chemiluminescence (ECL system, GE Healthcare). Protein bands were quantified using laser densitometry. Equal protein loading on membranes and complete transfer was confirmed by staining the gels and membranes with Coomassie Blue. To make the pooling of data from different immunoblots possible, the relative density of each band was normalized against the internal standard analyzed on each blot.

### Transfection of small interfering RNA

B16 cells were transfected with 50 nM Nrf2-annealed siRNA (Life Technologies) using Lipofectamine 2000 for 12 h according to the manufacturer's recommendations. The siRNA sequences targeted the following murine Nrf2 sequences: 5′-UGGAGCAAGACUUGGGCCACUUAAA-3′ and 5′ UUUAAGUGGCCCAAGUCUUGCUCCA-3′. Control experiments were performed using equivalent amounts of the corresponding sense oligonucleotides and scrambled oligonucleotides with the same base composition and a randomized sequence (5′-AUGGGCUAAAUCAUCCGCAAGAUGG-3′ and 5′-ACUGGCCAUUUCAGCUGAACCUUUG-3′).

### p53 antisense oligonucleotides

We used Cenersen (anti-p53-AS, Eleos Inc., Omaha, NE), a 20-mer antisense phosphorothioate oligonucleotide complementary to TP53 exon10 that can cleave TP53 mRNA through an RNase H-dependent mechanism, effectively down-regulating wild-type p53 expression *in vitro* and *in vivo*. Cells were transfected with Cenersen (5′-CCCTGCTCCCCCCTGGCTCC-3′; control oligonucleotide with Cenersen-reversed sequence (5′-CCTCGGTCCCCCCTCGTCCC-3′) or a scrambled sequence (5′CCTTCGGCCCTCCCCGCCCT-3′) for 48 h using Lipofectamine RNAiMAX (Life Technologies) according to the manufacturer's protocol.

### Isolation and culture of hepatic sinusoidal endothelium

Hepatic sinusoidal endothelium (HSE) from syngenic male C57BL/6J mice was separated in a 17.5% (w/v) metrizamide gradient and identified as previously described [30]. HSE cultures were established and maintained in pyrogen-free DMEM supplemented as described above for the B16 cells. Differential adhesion of endothelial cells to the collagen matrix and washing allowed complete elimination of other sinusoidal cell types (i.e., Kupffer, stellate, lymphocytes) from the culture flasks [28].

### B16-F10-endothelial cell adhesion and cytotoxicity assays

B16-F10 cells were loaded with 2′,7′-bis(2-carboxyethyl)-5,6-carboxyfluorescein acetoxymethyl ester (BCECF-AM, Life Technologies) (10^6^ cells were incubated in 1 ml of HEPES buffered DMEM containing 50 µg of BCECF-AM and 5 µl of Me_2_SO for 20 min at 37°C). Further cell processing and assays were performed as previously described [28]. The number of adhering tumor cells was quantified by arbitrary fluorescence units using a Fluoroskan Ascent FL (Labsystems, Manchester, UK) based on the initial number of B16-F10 cells added to the HSE culture [28]. Damage to B16-F10 cells during their *in vitro* adhesion to the HSE was measured as previously described [28] using tumor cells loaded with calcein-AM (Life Technologies).

### Measurement of H_2_O_2_, nitrite, and nitrate

Measurement of H_2_O_2_ based on the H_2_O_2_/horseradish peroxidase-dependent oxidation of homovanillic acid (3-methoxy-4-hydroxyphenylacetic acid) to a highly fluorescent dimer (2,2-dihydroxydiphenyl-5,5-diacetic acid) and flow cytometric determination of O_2_^−^ generation were performed as previously described (11). Nitrite and nitrate determinations were performed as previously described [30] and based on the methodology of Braman and Hendrix [31]. Total NO_x_ (NO_2_ **^–^** plus NO_3_ **^–^**) was determined by monitoring NO evolution from a measured sample placed into a boiling VCl_3_/HCl solution (which will reduce both NO_2_ **^–^** and NO_3_ **^–^** to NO). Quantitation was accomplished using a standard curve made up of known amounts of NO_2_ **^–^** and NO_3_ **^–^**.

### *In vivo* microscopy

Metastatic cell dynamics within the liver were examined as previously described [32] using calcein-AM-labeled B16-F10 cells. The total number of calcein-AM-labeled cells per hepatic lobule was recorded in 10 different lobules per liver at 15-min intervals and for a 6-h period. Cells were scored as ‘‘intact’’ non-damaged cells (round bright fluorescent cells with a well-delineated profile and no fluorescence diffusion from the cytoplasm to their neighboring hepatic tissue) or damaged (irregularly shaped fluorescent cells with diffuse fluorescence around them, staining the hepatic tissue). The microscope was an Eclipse E600FN, providing transillumination or epi-illumination, and equipped for video microscopy using a digital DXM 1200 camera (Nikon, Tokyo, Japan).

### Expression of results and statistical analysis

Data are presented as the means ± S.D. for the indicated number of different experiments. Statistical analyses were performed using Student's t test, and p<0.05 was considered significant.

## Results

### Effect of glucocorticoid receptor knockdown on the GSH content of metastatic B16 melanoma cells

In our studies the following B16 cell variants were used (see under Materials and Methods for experimental details): a) highly metastatic B16-F10 (ATCC); b) iB16 (cultured B16-F10, inoculated into mice, and isolated from hepatic or pulmonary metastatic foci or subcutaneous tumors); c) B16-F10-shGCR and iB16-shGCR (GCR knockdown cell variants).

**Figure 1:**
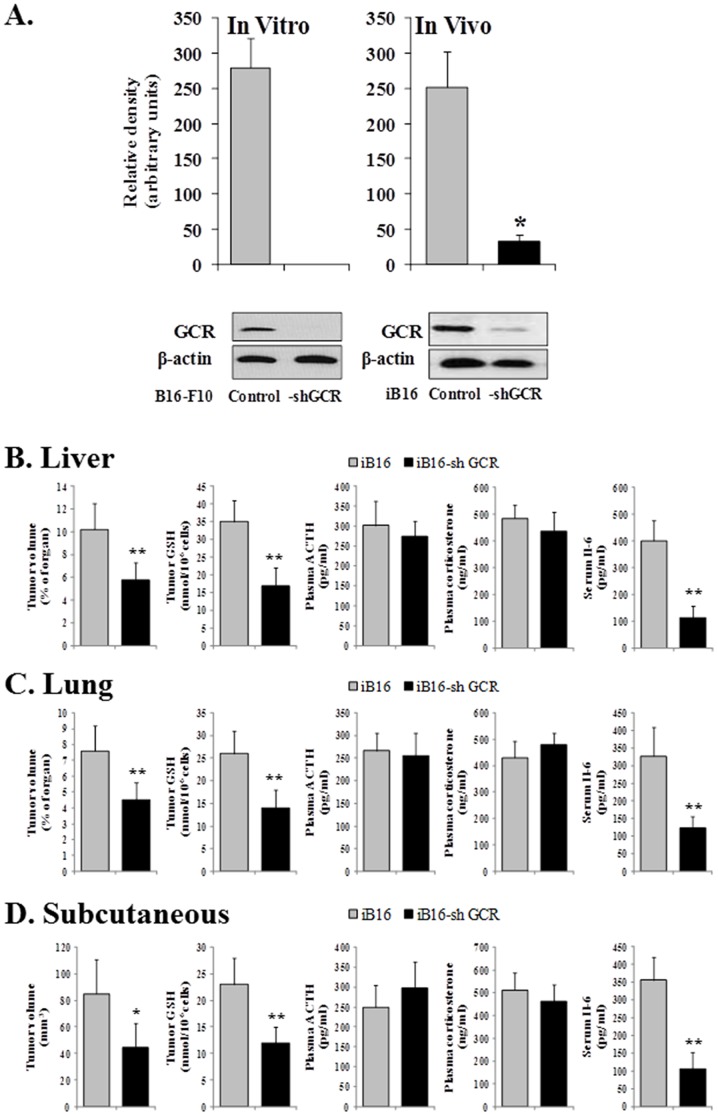
Glucocorticoid receptor knockdown and GSH content in B16 melanoma cell subsets; and plasma corticosterone, ACTH, and IL-6 levels during melanoma growth *in vivo*. (A) GCR levels were measured by Western blot in control metastatic iB16 melanoma cells isolated from the liver and their equivalents stably expressing GCR-shRNA. Similar blots were run for B16-F10 and B16-F10-shGCR growing *in vitro*. Each lane in the blots corresponds to an individual representative animal in the indicated group. The relative density of each band was normalized against the internal standard (β-actin) on each blot (n = 4–5 in all cases) and expressed as relative changes in arbitrary densitometry units. Results obtained in cells transfected with lentiviral vector not harboring any gene (negative control) were not different from control values (not shown). *p<0.01 *versus* iB16 cells. *In vivo* experiments show data obtained after 7 days of inoculation. *In vitro* experiments show results obtained in cells cultured for 72h. (B–D)Blood was collected from the tail vein during a 24-h period starting 7 days after tumor inoculation, and peak plasma levels of corticosterone and ACTH (6 h and 12 h, circadian time, respectively) measured. Melanoma cells were isolated before GSH determination. Tumor volume and GSH levels were measured 8 days after inoculation. Data are mean values ± S.D. of 7–8 different animals. *p<0.05, **p<0.01 *versus* controls.

Metastatic iB16-shGCR cells, isolated from metastatic foci growing in the liver, exhibited a significant decrease in GCR levels on Western blot compared to control iB16 cells. Similar results were observed in B16-F10-shGCR cells compared to control B16-F10 cells *in vitro* (Fig. 1A), or in iB16-shGCR cells growing in the lungs (results not shown).

The effect of GCR knockdown on tumor growth and GSH content in cancer cells growing at different sites was studied. GSH levels were significantly higher in metastatic iB16 cells compared to iB16-shGCR cells in liver and lung foci; a similar pattern was found in melanoma cells inoculated subcutaneously (Fig. 1B–D). Tumor growth decreased in all iB16-shGCR cancer cells compared to controls (Fig. 1B–D). Plasma levels of ACTH and corticosterone (the main circulating glucocorticoid in rodents) [33] were similar in all malignant cell types (control or iB16-shGCR), whereas circulating levels of IL-6 decreased in mice bearing iB16-shGCR cancer cells (Fig. 1B–D).

### Effect of glucocorticoids on GSH synthesis and efflux in metastatic B16 melanoma cells

**Figure 2.Effect:**
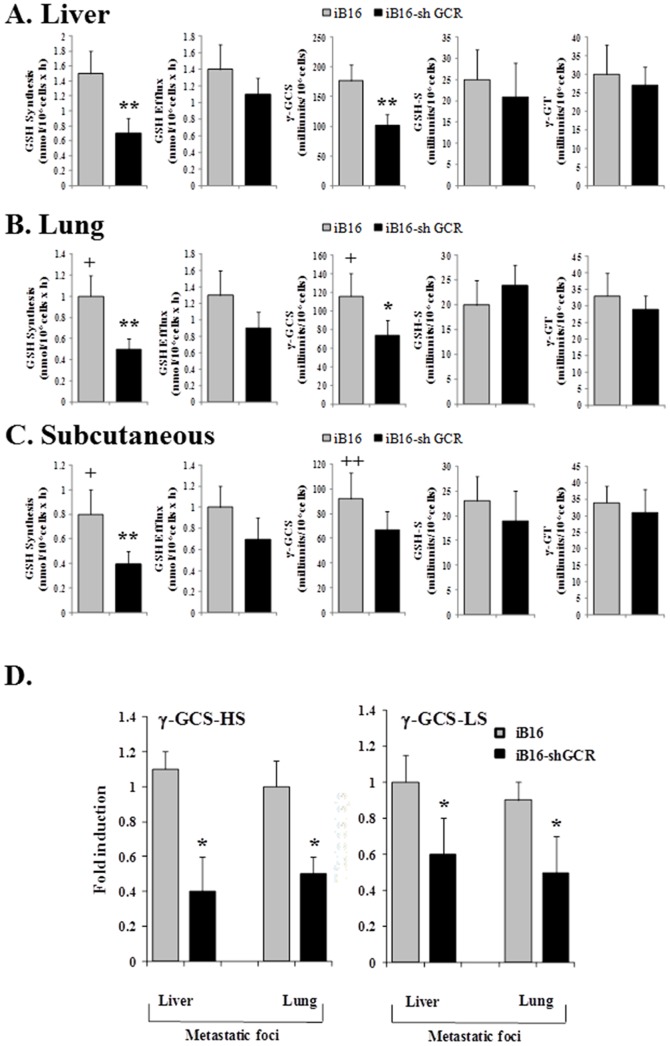
of glucocorticoid receptor knockdown on the rates of GSH synthesis and efflux in iB16 melanoma cells. (A–C) Melanoma cells were isolated from the liver or lungs 7 days after inoculation and from subcutaneous tumors 14 days after inoculation for culture. Glutathione efflux corresponded to GSH because GSSG was, in all conditions, 1–2% of the total glutathione found in the extracellular space (not shown). To prevent degradation of the GSH accumulated in the extracellular space, γ-GT was blocked by adding 10 µM acivicin to the culture medium 2 h before measuring efflux. Enzyme activities were measured 22 h after seeding. Results obtained in iB16 cells transfected with lentiviral vector not harboring any gene (negative control) were not different from control values (not shown). Data are mean values ± S.D. (n = 9–10 in all cases). *p<0.05,**p<0.01*versus* iB16 controls. +p<0.05, ++p<0.01 *versus* melanoma cells isolated from liver metastases. (D) γ-GCS-HS and γ-GCS-LS expression was determined in cells cultured for 24h (previously isolated from *in vivo* tumors). Data, expressed as a fold change, show mean values ± S.D. from 5 to 6 different experiments. *p<0.01 *versus* iB16 cells.

In order to investigate the mechanism underlying the effect of GCR knockdown on GSH levels, we measured the rates of GSH synthesis and efflux in different melanoma cell subsets. Cells were isolated from metastatic foci or tumors grown subcutaneously. GSH synthesis was significantly lower in tumor cells growing in the lung or subcutaneously compared to the liver (Fig. 2A–C). However, as shown in Fig. 2, the rate of GSH synthesis (measured *in vitro* in isolated cells and in the presence of amino acid precursors, see the caption) was significantly lower in iB16-shGCR cells than in iB16 controls for all tumor locations. These findings correlate with similar differences in γ-GCS activity (Fig. 2A–C), the rate-limiting step in GSH synthesis [34], and GSH content (Fig. 1B–D). γ-GCS is a heterodimer consisting of catalytic (γ-GCS-HS, 73 kDa) and regulatory (γ-GCS-LS, 31 kDa) subunits[35]. As shown in Fig. 2D, the decrease in γ-GCS activity in iB16-shGCR metastatic cells was accompanied by a decreased in the expression of both γ-GCS-HS and γ-GCS-LS. GSH-S and γ-GT activities were similar in all cell subsets (Fig. 2A–C). Rates of GSH efflux were not significantly different when iB16-shGCR cells and iB16 cells (at each tumor localization) were compared, or when each cell subset growing in the lungs or subcutaneously were compared with their corresponding counterparts growing in the liver (Fig. 2A–C). Therefore these results suggest that the decrease in GSH content in iB16-shGCR cells, compared to iB16 controls, is due to lower rates of GSH synthesis and not to changes in the rate of GSH release or breakdown.

### Glucocorticoids and activation of Nrf2 in metastatic B16 melanoma cells

**Figure 3:**
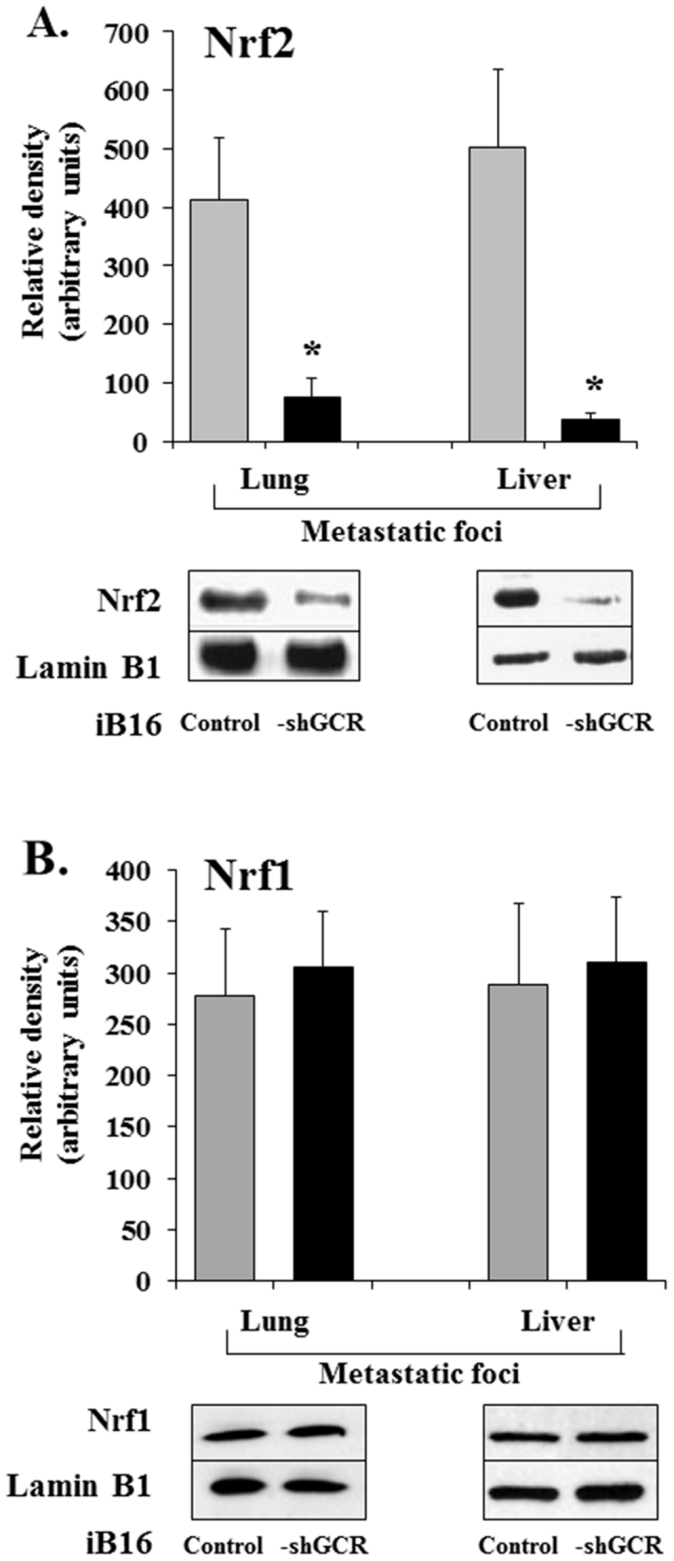
Glucocorticoid receptor knockdown is associated with a decrease in nuclear Nrf2. iB16 or iB16-shGCR cells were isolated from metastatic foci growing in the liver or lung and nuclear accumulation of Nrf1 and Nrf2 measured by Western blotting. Results obtained in iB16 cells transfected with lentiviral vector not harboring any gene (negative control) were not different from control values (not shown). Data show mean values ± S.D. from 5 to 6 different experiments. *p<0.01 *versus* iB16 cells.

The human and murine γ-GCS-HS and γ-GCS-LS promoter regions share similar regulatory mechanisms [36]. Nrf1 and Nrf2 transcription factors are central mediators in the expression of the γ-GCS subunits in response to oxidative stress and through activation of antioxidant/electrophile response elements (ARE/EpRE) [36]. When activated by oxidative stress Nrf1 and Nrf2 form obligate heterodimers with other factors, such as small Maf and Jun proteins, to bind to ARE/EpRE and regulate the transcription of oxidative stress-related genes [37]. Increased expression of γ-GCS-HS and γ-GCS-LS genes has been associated with an increase in the binding of Nrf1 and Nrf2 to ARE/EpRE in the promoters of these genes [38], [39]. Thus, because glucocorticoids increase ROS generation in metastatic B16 melanoma cells [6], we investigated whether the decrease in γ-GCS activity in iB16-shGCR metastatic cells is associated with changes in nuclear Nrf1 and/or Nrf2. As shown in Fig. 3, nuclear Nrf2, but not Nrf1, decreased in iB16-shGCR cells isolated from lung or liver metastatic foci compared to control iB16 cells.

To further prove the involvement of Nrf2 in regulating γ-GCS activity in metastatic cells, we used anti-Nrf2-siRNA to directly interfere with Nrf2 expression. As shown in Table 1, transfection of iB16 cells with anti-Nrf2-siRNA decreased Nrf2 levels as well as γ-GCS activity and GSH levels. However, although anti-Nrf2-siRNA transfection decreased H_2_O_2_ generation in iB16 cells, O_2_^−^ production remained close to control values (Table 1).

**Table 1:** ROS, Nrf2 and GSH levels, and γ-GCS activity in iB16 and iB16-shGCR cells isolated from metastatic foci.

	Metastatic cells		
Parameter	iB16	iB16-shGCR	iB16 +anti-Nrf2-siRNA
**H_2_O_2_** (nmol/10^6^cells x min)	1.45±0.30	0.63±0.18**	0.37±0.12**
**O_2_** (ΔFL1, AU)	3.74±0.57	1.71±0.36**	3.09±0.33*
**Nrf2** (relative density, AU)	272 ±53	134±37**	32±8**
**γ-GCS** (milliunits/10^6^cells)	155±29	83±17**	42±15**
**Tumor GSH** (nmol/10^6^cells)	30±4	15±3**	10±2**

Melanoma cells were isolated from the liver 7 days after inoculation, cultured, and transfected with anti-Nrf2-siRNA. H_2_O_2_ and O_2_^−^generation, γ-GCS activity, and GSH levels were measured 48 h after seeding. Nrf2 levels (Western blotting) were measured 24 h after seeding. AU, arbitrary units. Data are mean values ± S.D. (n = 6–7 in all cases). *p<0.05,**p<0.01 *versus* iB16 controls. Results obtained in cells transfected with control Nrf2 sense or scrambled oligonucleotides were not significantly different from those obtained in cells cultured in the absence of anti-Nrf2-siRNA (not shown).

**Figure 4:**
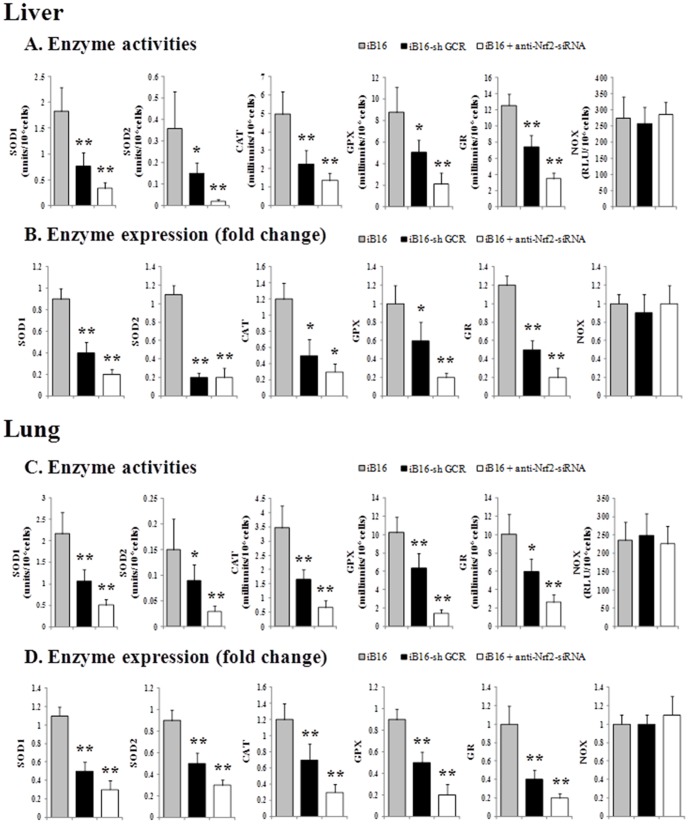
Antioxidant enzyme activities and expression in different metastatic B16 melanoma cell subsets. (A) and (C) Enzyme activities were measured in metastatic cell subsets isolated from growing liver or lung foci 7 days after inoculation and cultured for 48 h. The enzyme activities before inoculation in B16-F10 cells cultured for 48 h were: SOD1, 1.51±0.33 units/10^6^cells; SOD2, 0.14±0.05 units/10^6^cells; CAT, 4.22±1.05 milliunits/10^6^cells; GPX, 7.51±1.63 milliunits/10^6^cells; GR, 6.58±2.04 milliunits/10^6^cells; and NOX, 183±42 RLU/10^6^cells (n = 6 in all cases). iB16 cells were transfected *in vitro* with anti-Nrf2-siRNA as in Table 1. RLU, relative light units. Data are mean values ± S.D. (n = 5–6 in all cases). *p<0.05, **p<0.01 *versus* iB16 controls. Enzyme activities measured in iB16 cells transfected with Nrf2 sense or scrambled oligonucleotides were not significantly different from control values (not shown). (B) and (D)Melanoma cells isolated from liver or lung metastatic foci 7 days after inoculation were cultured for 48 h. Results obtained in iB16 cells transfected with lentiviral vector not harboring any gene (negative control) were not different from control values (not shown).Data from quantitative RT-PCR are expressed as mean fold change ± S.D. (n = 6 in all cases). *p<0.05, **p<0.01 *versus* iB16 controls. Enzyme expression measured in iB16 cells transfected with Nrf2 sense or scrambled oligonucleotides was not significantly different from control values (not shown).

In addition to γ-GCS, Nrf2 also controls the expression of different antioxidant enzymes [40]. To further analyze the molecular mechanisms underlying the effects of GCR knockdown in metastatic cells, we measured the activity of different oxidative stress-related enzymes. As shown in Fig. 4A and C, GCR knockdown decreased SOD1, SOD2, CAT, GPX, and GR, but not NOX, activities in iB16 cells isolated from different metastatic foci. Treatment with anti-Nrf2-siRNA also decreased the activity of SOD1, SOD2, CAT, GPX, and GR in iB16 cells. SOD1 decreased to approximately 18% and 23% of control values in the liver and lung, respectively, whereas SOD2 decreased to 5% and 20% of control values in the liver and lung, respectively (Fig. 4 A and C). Although there is a strong Nrf2-dependence, SOD1 and SOD2 activities in B16-F10 cells growing *in vitro* were lower than those measured in the same cells under *in vivo* conditions (see caption, Fig. 4).Therefore the *in vivo*-related increase in SOD2 is higher than that of SOD1, suggesting that SOD2 may be more responsive to the pro-oxidant metastatic microenvironment [2], [3]. Data corresponding to enzyme activities (Fig. 4A and C) correlated with similar experiments performed in parallel to measure the expression of these enzymes (Fig. 4B and D).

However, transfection with anti-Nrf2-siRNA did not affect NOX activity or expression (Fig. 4), which may explain the maintenance of a high rate of O_2_^−^ production (Table 1). In iB16 cells transfected with anti-Nrf2-siRNA and cultured in the presence of 30 µM VAS3497 (a triazolo pyrimidine that specifically inhibits NOX activities) [27], O_2_^−^ production (FL1) decreased to 1.04±0.26 (n = 5, p<0.01 compared to control iB16 cells, Table 1). This finding suggests that NOX activity is a primary Nrf2-independent source of O_2_^−^ in metastatic iB16 cells. The specific NOX isoforms involved and their transcriptional regulation in melanoma, as well as in other cancer cells with metastatic potential, are still unknown [41].

### p53 suppresses the Nrf2-dependent transcription of antioxidant enzymes

Evidence obtained from cancer patients and cell lines suggests that Nrf2 is highly active in a variety of human cancers and associated with aggressiveness [42]. In parallel with the Nrf2-dependent antioxidant response, cells can counteract the consequences of oxidative stress by attempting to repair the ROS- and/or electrophile-induced damage [2]. The tumor suppressor p53 is activated by DNA damage and regulates the expression of many target genes, thus leading to cell cycle arrest to allow time for the repair of DNA damage [43]. In addition, p53 plays a fundamental role in the induction of apoptosis in cells with unrepaired DNA damage [43]. Thus, cross-talk likely occurs between the Nrf2- and p53-induced responses. Studies have reported that p53 can interfere with the Nrf2-dependent transcription of ARE-containing promoters [44]. Nevertheless, in approximately half of all human cancers, particularly highly aggressive and metastatic cancers, the p53 protein is reduced, lost, or mutated [45], [46]. Hypothetically, this molecular limitation could be, at least part of, the underlying mechanism explaining the strong Nrf2-dependence of different antioxidant enzyme activities found in metastatic B16 cells.

**Figure 5:**
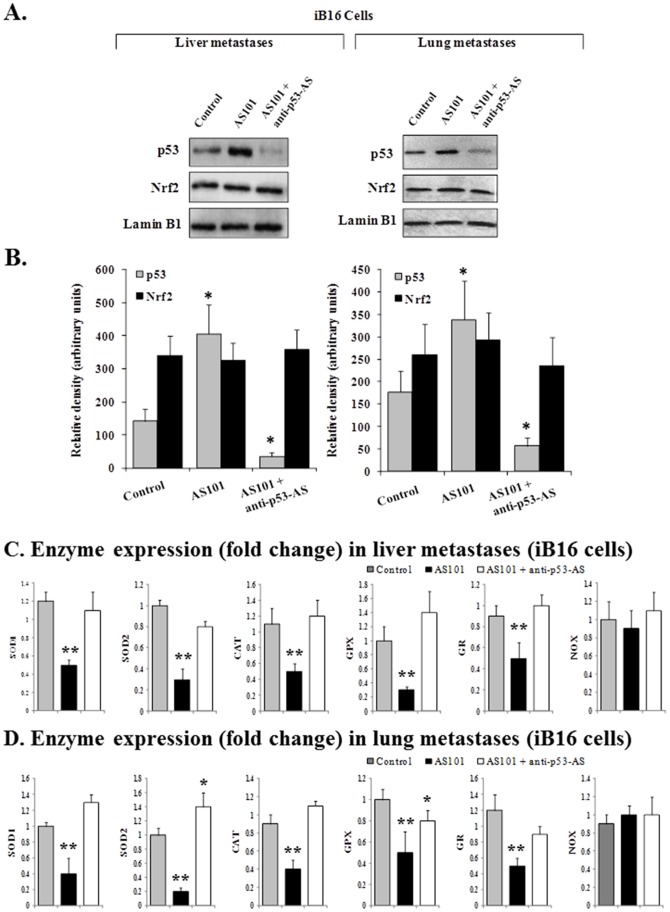
Effect of AS101 and anti-p53 antisense oligonucleotides on nuclear p53 and Nrf2 levels, and expression of oxidative stress-related enzymes in metastatic melanoma cell subsets. (A) and (B) Melanoma cells isolated 7 days after inoculation were cultured for 48 h. Western blot (A), protein band quantification (B), and data pooling (n = 5–6 in all cases) were performed as in Fig. 1. AS101 (0.1 µg/ml) was added to the culture medium 2 h after seeding. Oligonucleotides (50 nM) were added 2h and 24 h after seeding as 1∶1 complexes with the Lipofectamine RNAiMAX reagent. Data are mean values ± S.D. (n = 4–5 in all cases). *p<0.01 *versus* controls.(C) and (D)Melanoma cells isolated from liver or lung metastatic foci 7 days after inoculation were cultured for 48 h. Data from quantitative RT-PCR are expressed as mean fold change ± S.D. (n = 5–6 in all cases). *p<0.05, **p<0.01 *versus* controls.(A–D) Results obtained in iB16 cells transfected with p53 sense or scrambled oligonucleotides were not significantly different from those obtained in controls or cells incubated with AS101 alone (not shown).

We explored this possibility using ammonium trichloro (dioxoethylene-0,0′) tellurate (AS101), an immunomodulator first synthesized at Bar-Ilan University (Ramat-Gan, Israel) that increases expression of wild-type p53 in B16-F10 melanoma cells [45]. As shown in Fig. 5, AS101-induced up-regulation of p53 (Fig. 5A and B) in cultured iB16 melanoma cells caused a decrease in antioxidant enzyme expression (Fig. 5C and D) but did not affect nuclear levels of Nrf2 (Fig. 5A and B).This effect was reversed by using anti-p53 antisense oligonucleotides (Fig. 5), indicating that p53 can influence Nrf-2-dependent antioxidant enzyme expression. Moreover, AS101-induced upregulation of p53 levels also associated with a decrease in γ-GCS-HS and γ-GCS-LS expression, γ-GCS activity and, consequently, in GSH levels in metastatic cells. Thus further supporting the role of p53 in downstream targets of Nrf2 (Table 2).

**Table 2:** Effect of AS101 and anti-p53 antisense oligonucleotides on γ-GCS activity and expression and on GSH levels in metastatic melanoma cell subsets.

	Metastases					
	Liver			Lung		
	Control	AS101	AS101 + anti-p53-AS	Control	AS101	AS101 + anti-p53-AS
**γ-GCS** (milliunits/10^6^ cells)	163±32	93±17*	150±26	104±20	50±21*	89±18
**Enzyme expression** (fold induction)						
** γ-GCS-HS**	1.0±0.1	0.3±0.2*	0.9±0.3	1.05±0.2	0.4±0.2*	1.0±0.3
** γ-GCS-LS**	1.1±0.2	0.5±0.1*	0.9±0.1	1.1±0.2	0.6±0.1*	0.9±0.2
**GSH** (nmol/10^6^ cells)	38±7	21±6*	33±4	23±6	13±5*	24±5

Measurements and treatments were performed in isolated metastatic cells as indicated in the legend to Fig. 5. Control experiments on p53 and Nrf2 levels were similar to those obtained in Fig. 5 A (not shown). Results obtained in iB16 cells transfected with p53 sense or scrambled oligonucleotides were not significantly different from those obtained in controls or cells incubated with AS101 alone (not shown). Data are mean values ± S.D. (n = 4–5 in all cases). *p<0.01 *versus* controls.

### Effect of glucocorticoid receptor knockdown on the sensitivity of metastatic B16 melanoma cells to vascular endothelium-induced tumor cytotoxicity

The arrest of B16 melanoma cells in the liver microvasculature induces endogenous NO and H_2_O_2_ release, leading to intrasinusoidal tumor cell killing [30]. Nevertheless, a high percentage of metastatic B16 cells with high GSH content manage to survive the combined nitrosative and oxidative attack elicited by the vascular endothelium [30]. Theoretically, the GCR knockdown-induced decrease in the antioxidant protection of metastatic cells could increase their sensitivity to vascular endothelium-induced cytotoxicity. We assayed this possibility first *in vitro*. As previously described [32], primary cultures of freshly isolated syngenic HSE were used to reproduce the adhesion of B16 cells to the liver sinusoidal wall *in vitro*. As shown in Table 3, B16-F10 cells cultured to low density (high GSH content) [30] and co-cultured with HSE cells exhibited a small 17% decrease in viability during the interaction with HSE cells. However, L-buthionine (SR)-sulphoximine (BSO), the specific GSH synthesis inhibitor [35], induced GSH depletion and increased the loss of B16-F10 cell viability to 72% (Table 3). On the other hand, the viability of co-cultured iB16-shGCR cells isolated from solid subcutaneous tumors without previous metastatic dissemination and incubated in the presence of BSO decreased by 85% (Table 3). This result is not surprising because the GCR knockdown-associated decrease in antioxidant enzyme protection (Fig. 4) could increase the sensitivity of iB16-shGCR to endothelium-derived oxidative/nitrosative stress. The total amount of NOx and H_2_O_2_ that accumulated in the culture medium (mainly released by the endothelium) [30], during the first 2h of interaction between B16-F10 and HSE cells, was of 7.4±1.4 and 65±17 nmol/10^6^ cells respectively. These values were not significantly different from the interaction of iB16-shGCR and HSE cells (n = 5).

**Table 3:** Effect of GR knockdown and GSH depletion on the *in vitro* interaction between B16 melanoma cells and the vascular endothelium.

		B16-F10 + HSE		iB16-shGCR (subcutaneous) +HSE	
	Melanoma cell pretreatment with BSO…	-	+	-	+
Tumor GSH before co-culture (nmol/10^6^cells)		31±6	12±3*	16±3*	9±2*
Tumor cytotoxicity (%)		17±4	72±14*	65±12*	85±14*

HSE cells (2.5×10^5^cells/well) cultured for 24 h were co-cultured with B16-F10 or iB16-shGCR cells (5.0×10^5^cells/well; pre-cultured for 24 h). Twenty minutes after the addition of tumor cells to the HSE, the plates were washed as described in Materials and Methods. The ratio of tumor cells adhering to the HSE was 1∶1. TNF-α (100 units/ml) and IFN-γ (50 units/ml), which were used as potent activators of NO and H_2_O_2_ generation by the HSE, were added to the co-cultures when all tumor cells present were attached to the HSE. In endothelium-induced B16-F10/iB16-shGCR cytotoxicity assays, tumor cytotoxicity (expressed as the % of tumor cells that lost viability within the 3–6-h incubation period) was determined after 6 h of incubation. During the 6-h incubation period, the percentage of HSE cell viability was 98–99% in all cases. When adding cytokines to cultured tumor cells alone, no cytostatic or cytotoxic effects were observed within the next 6 h. During the first 2-hincubation period, both HSE and B16-F10 or iB16-shGCR cells maintained >95% viability (data not shown). Where indicated, B16-F10 or iB16-shGCR cells were incubated for 24 h with BSO (0.5 mM) before co-culturing with endothelial cells. Pretreatment of B16-F10 cells with BSO did not significantly affect control values for tumor cell adhesion. Data are means ± S.D. for 5–6 independent experiments. *p<0.01 *versus* B16-F10 + HSE controls in the absence of BSO.

**Figure 6:**
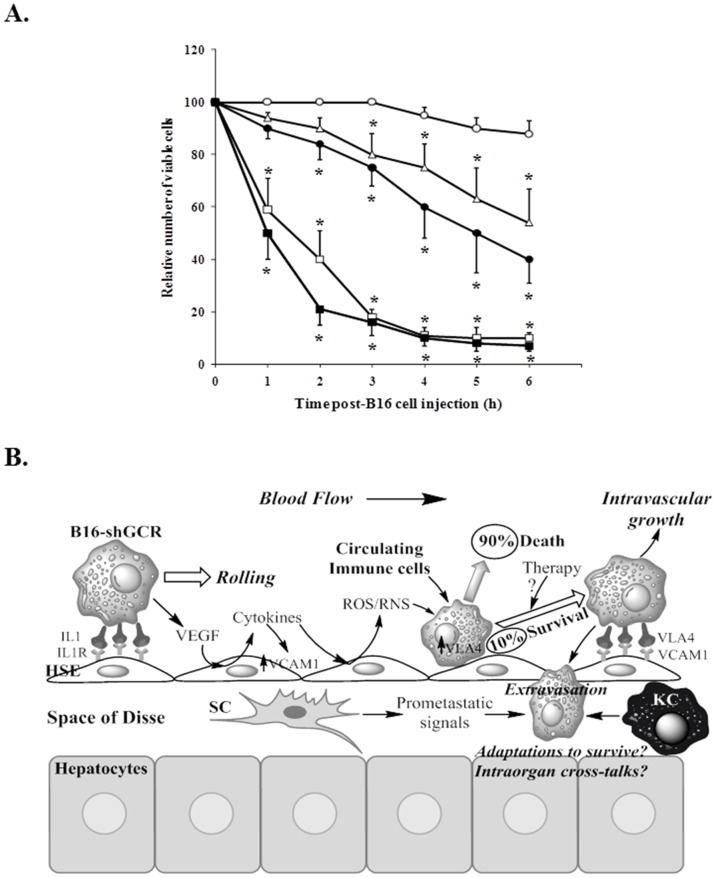
Effect of glucocorticoid receptor knockdown and GSH depletion on the invasive activity of B16 melanoma cells in the liver. (A) *In vivo* video microscopic study of the viability of intraportally injected B16 melanoma cell subsets arrested in the mouse liver microvasculature. B16-F10 (○), B16-F10 pre-cultured for 24 h in the presence of 0.5 mM BSO (•), iB16-shGCR isolated from solid tumors growing in the foot pad (□), iB16-shGCR pre-cultured for 24 h in the presence of 0.5 mM BSO (▪), and iB16-shGCR pre-cultured for 24 h in the presence of 1.0 mM GSH ester (Δ). The average number of arrested B16 cells per hepatic lobule was similar independently of the cell subset considered. Results obtained in iB16 cells transfected with lentiviral vector not harboring any gene (negative control) were not different from control values (not shown). Data are mean values ± S.D. from 4 to 5 different experiments. *p<0.01 *versus* B16-F10 controls. (B) In a first step, metastatic B16 cells establish a weak molecular bridge (docking) with the vascular endothelium. Metastatic growth factors induce endothelial cytokine release and, consequently, generation of high ROS and RNS levels that, in cooperation with the immune system, cause tumor cytoxicity in up to 90% of all attached B16-shGCR cells. Subsequent rolling facilitates locking through very late antigen 4 (VLA4) and intercellular adhesion molecule 1 (VCAM1). Cancer cells attached to the endothelium of pre-capillary arterioles or capillaries may follow two mechanisms of extravasation: a) migration through vessel fenestrae and/or b) intravascular proliferation followed by vessel rupture and microinflammation. Invading cancer cells will form micrometastases within the normal lobular hepatic architecture via a mechanism regulated by cross-talk with the stroma and multiple microenvironment-related, and possibly also systemic, molecular signals. Activation of angiogenesis will facilitate metastatic growth and spread. The result of conventional/targeted therapy on the small percent of surviving metastatic cells or whether they adapt during invasion, generating more resistant cell subsets, are unanswered questions. VEGF, vascular endothelial growth factor; SC, stellate cell; KC, Kupffer cell.

Next, we assayed the interaction of B16 melanoma cells with the vascular endothelium *in vivo* as a critical step previous to tissue/organ invasion. We used an experimental setup specifically designed for *in vivo* observation of the liver microcirculation. As shown previously [32], acute liver inflammation was induced by a single i.v. injection of 0.5 mg/kg lipopolysaccharide 6 h before B16 melanoma cell injection. Using previously described methodology for assays in this and other experimental tumors [32], calcein-labeled B16 cells, which present a green fluorescent cytoplasm, were arrested within a few seconds after intraportal injection. As shown in Fig. 6A, the relative number of intact B16 melanoma cells arrested within the hepatic microvasculature progressively decreased for a 6-h period after inoculation to approximately 88% in control B16-F10 cells (32±4 nmol GSH/10^6^ cells before injection), 40% in B16-F10 cells pretreated *in vitro* with BSO (11±2 nmol GSH/10^6^ cells before tumor cell injection, p<0.01 vs. control), 10% in iB16-shGCR cells (14±3 nmol GSH/10^6^ cells before injection, p<0.01 vs. control), 7% in iB16-shGCR cells pretreated *in vitro* with BSO (11±2 nmol GSH/10^6^ cells before injection, p<0.01 vs. control), and 54% in iB16-shGCR cells pretreated *in vitro* with GSH ester (which enters the cell and delivers free GSH) (16) (46±7 nmol GSH/10^6^ cells before injection, p<0.01 vs. control; n = 5–6 in all cases). From these data we can conclude that: a) BSO-induced GSH depletion decreases B16-F10 cell viability upon interaction with the HSE, and b) iB16-shGCR cells with low GSH content also lose viability, but to a much greater extent. The lower activity of different antioxidant enzymes increases the sensitivity of these metastatic cells to the cytotoxic effect of ROS/reactive nitrogen species (RNS) released by the endothelium. Nevertheless, 10% of iB16-shGCR cells remain viable and potentially capable of invading the organ as suggested by the rapid growth rate indicated in Fig. 1. Moreover, the exceptional resistance of this metastatic cell subset may imply that these cells have developed the ability to survive and/or adapt towards a higher resistance phenotype *in vivo*. Fig. 6B schematically summarizes the molecular events that occur during B16-F10 melanoma cell attachment to the hepatic endothelial cells and subsequent tissue invasion. This figure includes already known mechanisms, our present observations, and some key questions. Studies on these potential survival/adaptation mechanisms are now underway in our lab.

## Discussion

Low levels of both ROS and RNS are continuously produced in mammalian cells, and they play important physiological roles [47]. However, when the amount of ROS/RNS exceeds the capacity of the antioxidant machinery, the resulting oxidative/nitrosative stress may induce irreversible damages in all cellular macromolecules [48].

Cancer cells that survive the circulatory system and reach different organs/tissues interact with the vascular endothelium to begin secondary colonization [48]. The interaction of cancer and endothelial cells in capillary beds, a critical step in the initiation of metastasis, involves mechanical contact and transient adhesion. This interaction initiates a cascade of activation pathways involving cytokines, growth factors, bioactive lipids, ROS, and RNS produced by cancer cells and the endothelium [48]. The interaction between murine B16 melanoma and the HSE involves mannose receptor–mediated melanoma cell attachment to the HSE, which causes subsequent proinflammatory cytokine release (i.e., TNF-α, IL-1β, and IL-18), as well as VCAM-1–dependent adherence that reinforces or locks the initial intercellular binding [2] (see Fig. 6B). B16-F10 cells express high levels of the integrin VLA-4, the ligand for VCAM-1 on activated endothelial cells [49]. Upon exposure to cytokines released during the interaction with metastatic cells, endothelial cells undergo profound alterations in their function that involve changes in gene expression, *de novo* protein synthesis, and the production of cytotoxic ROS and RNS [30], [50] (Fig. 6B). We showed that, by inhibiting NO production using HSE cells isolated from endothelial nitric oxide synthetase (eNOS)-deficient mice or L-NAME (an inhibitor of all NOS activities), H_2_O_2_ released by the HSE does not induce tumor cytotoxicity [30]. However, NO was tumoricidal in the presence of H_2_O_2_ because the addition of exogenous CAT, which eliminates H_2_O_2_ released into the extracellular medium, significantly decreased tumor cytotoxicity [30]. We found that a major portion of the effect requires the presence of trace metals capable of generating highly oxidant radicals, such as ^•^OH and ^–^OONO [30]. Immune cells are also present in the metastatic microenvironment. Both innate and adaptive immunity participates in anti-tumor effects, including the activity of natural killer cells, natural killer T cells, macrophages, neutrophils, eosinophils, complement proteins, various cytokines, specific antibodies, and specific T cytotoxic cells. Upon activation, macrophages and neutrophils are able to kill tumor cells, but they can also release tumoricidal ROS/RNS, and angiogenic and immunosuppressive substances [51]. In this complex scenario, the antioxidant defenses of the metastatic cells appear to be important for their survival and invasive activity. Different primary observations support this hypothesis in the B16-F10 model: B16 cells pretreated *in vitro* with the lipophilic antioxidant tocopherol (vitamin E) exhibit increased survival in the hepatic sinusoids [52]; an increase in B16 cell GSH content upon hydroxyurea treatment also transiently increases metastasis [53]; capillary survival decreases in GSH-depleted B16 cells [32]; and B16 cells with high GSH content exhibit higher metastatic activity in the liver than those with lower GSH content [17].

Recently we observed that pathophysiological levels of corticosterone induce cell death, mainly mitochondria-dependent apoptosis, in metastatic B16-F10 cells with low GSH content [6]. Redox-sensitive cysteine residues sense and transduce changes in cellular redox status caused by the generation of ROS, RNS, reactive electrophilic species, and the presence of oxidized thiols [54]. The oxidation of such cysteines is converted into signals that control cell regulatory pathways and induce gene expression [54]. Redox-sensitive transcription factors, including p53, NF-κB, and the FoxO family, can directly regulate the expression of different Bcl-2 family members [55]. Moreover, accumulating evidence suggests that GSH may play important roles in cell signaling [56]. Therefore, by directly regulating the activity of redox-sensitive transcription factors and/or by decreasing ROS, GSH levels may affect the expression of proteins involved in regulating, for example, apoptosis. In the present study, we observed that GSH levels were significantly higher in metastatic iB16 cells than in iB16-shGCR cells in both liver and lung foci as well as in solid growing tumors (Fig. 1 B–D). Thus suggesting that glucocorticoids may also favor the maintenance of GSH levels. We investigated this apparent biological paradox and found that the decrease in GSH content in iB16-shGCR cells, compared to iB16 controls, was due to lower rates of (Nrf2-dependent) GSH synthesis and not to changes in the rate of GSH release or breakdown (Figs. 2 and 3). Cellular heterogeneity in *in vivo* tumors also implies the presence of cancer cells with different GSH content within the same tumor [2]. Therefore, pathophysiological levels of glucocorticoids may have opposite effects on metastatic cell subsets depending on their initial GSH content.

Our results (Fig. 1 B–D) confirm our previous observations in metastatic B16 melanoma-bearing mice that treatment with RU-486, a GCR blocker, induces a decrease in circulating IL-6 [6]. IL-6 activates the release of hepatic GSH and its interorgan transport to the growing cancer cells [5]. This mechanism is highly dependent on stress hormone (corticosterone and NORA) induced IL-6 expression and secretion by cancer cells [6]. Nevertheless, extracellular GSH, transported by the bloodstream to the growing tumor, must be degraded and then resynthesized within the cancer cell [2]. *In vivo,* iB16-shGCR melanoma cells have lower GSH levels than controls, indicating that glucocorticoids influence GSH metabolism in metastatic cells.

GCR knockdown in iB16 cells was also associated with a decrease in ROS generation (Table 1) and lower levels of different antioxidant enzyme activities without affecting the O_2_^−^-generating NOX activity (Fig. 4). Thus indicating that GCR knockdown down-regulates the antioxidant protection of metastatic cells. This down-regulation leads to an increase in the sensitivity of metastatic cells to the tumoricidal activity elicited by the vascular endothelium *in vitro* (Table 3) and *in vivo* (Fig. 6A). During the initial 6-h post-inoculation period, iB16-shGCR cells attached to the HSE lost 90% of their viability (compared with 12% in control B16-F10 cells) (Fig. 6 A). This dramatic GCR-dependent loss in metastatic cell viability may have important clinical and regulatory implications. First, three main cancer types are susceptible to glucocorticoid resistance (thus evading glucocorticoid-induced apoptotic effects), including acute lymphoblastic leukemia, osteosarcoma, and small-cell lung carcinoma [9]. However, most cancers have a glucocorticoid-sensitive phenotype and could be susceptible to treatment with a therapy targeting GCRs. Second, if combined with GSH-depleting strategies [2] and conventional/target oncotherapies, GCR antagonists could likely improve anticancer effects. For example, RU-486, a GCR antagonist, is used for the treatment of several cancers, including breast, ovarian, and prostate, and glaucoma [57], and it has been shown to sensitize renal carcinoma cells to TRAIL-induced apoptosis through up-regulation of DR5 and down-regulation of c-FLIP(L) and Bcl-2 [58]. Nevertheless, suppression of the Nrf2-dependent antioxidant response by glucocorticoids has been shown in human embryonic kidney-293 and rat hepatoma Reuber H4IIE cells *in vitro* [59]. Can this apparent biological paradox be explained? GCR knockdown decreases ROS generation in iB16 cells, and lower ROS levels are associated with a decrease in nuclear Nrf2 in metastatic cells (Fig. 3, Table 1), whereas acute oxidative stress and inflammation (as occurs in organs invaded by cancer) may also be associated with impaired activation of Nrf2 [60]. Therefore, the concentration of glucocorticoids and GCRs, and/or the fluctuating levels of ROS (and possibly RNS) may be determinant for metastatic cell survival *in vivo*. Within the tumor microenvironment, GCRs in cancer, stromal cells, and tumor-associated macrophages are activated by physiological agonists from circulating blood that are released following central nervous system-dependent circadian patterns [61], [62]. Furthermore, specific tissue/organ-derived factors that are still undefined may contribute to GCR expression by metastatic cells.

Moreover, wild-type p53 can physically interact with the GCR forming a complex that results in cytoplasmic sequestration of both p53 and GCR, thus repressing the GC-dependent transcriptional activity [63], [64]. Therefore drugs or oligonucleotides, that could specifically increase p53 levels in metastatic cells, would be of potential benefit for cancer therapy. In this sense the combined use of e.g. AS101 and RU-486 appears a reasonable option that should be explored.

It is also feasible that iB16-shGCR cells that survive the interaction with the vascular endothelium may activate other survival/defense mechanisms. Recent studies of the pro-apoptotic protein BIM, which is involved in the apoptosis of glucocorticoid-sensitive (CEM-C7) and -resistant (CEM-C1) acute lymphoblastic leukemia CEM cells, have shown that treatment with dexamethasone plus RU486 blocked apoptosis and BIM expression in CEM-C7 cells [65]. P38MAPK-blocking pharmacon SB203580 also significantly inhibits the up-regulation of BIM in CEM-C7 cells [65]. This evidence suggests that the absence of BIM up-regulation is one of the important mechanisms underlying glucocorticoid resistance, and glucocorticoid-GCR conjugation is indispensable in both glucocorticoid-induced apoptosis and BIM up-regulation. The p38 MAPK signaling pathway is also involved in this process. Interestingly, ROS have been reported to control the expression of Bcl-2 proteins by regulating their phosphorylation and ubiquitination [66]. Therefore, depending on the cancer cell type and conditions, the regulation of some pro-/anti-death Bcl-2 proteins may be influenced by GCR blockers and oxidative/nitrosative stress. Notably, Blc-2, in particular, can inhibit GSH efflux and, thus, favors GSH accumulation within the cancer cell [4]. This conclusion has experimental and clinical relevance as different Bcl-2 over-expressing melanomas have been observed to exhibit more aggressive behavior [67].

In conclusion, GCR knockdown decreases nuclear Nrf2, a master regulator of the antioxidant response, leading to a decrease in γ-GCS and other oxidative-stress-related enzyme activities in metastatic B16 cells. Decreased antioxidant protection causes an increase in the tumoricidal activity elicited by the vascular endothelium. Thus, GCR blockers, if used in combination with anticancer therapies, may possibly improve their effectiveness. The present results further support our previous proposal [6] indicating that metastatic cells utilize physiological neuroendocrine mechanisms to survive and grow.
